# Effects of camel hump fat, palm olein oil, and corn oil feed additives on plasma lipids and adipose tissues in rats

**DOI:** 10.3389/fnut.2025.1587579

**Published:** 2025-04-07

**Authors:** Shaheed Mohammed Alshaikhsaleh, Farag Ali Saleh, Mutlag Mohammed Al-Otaibi

**Affiliations:** Department of Food and Nutrition Sciences, Faculty of Agriculture and Food Sciences, King Faisal University, Al-Ahsa, Saudi Arabia

**Keywords:** adipose tissue, camel hump fat, corn oil, hyperlipidemia, obesity, palm olein oil

## Abstract

Hyperlipidemia is an important risk factor for cardiovascular disease and a leading cause of mortality and is often associated with obesity. Different types of fats and oils may have different effects on cardiovascular disease and obesity. This study investigated the effects of palm olein oil (PO), corn oil (CO), and camel hump fat (CHF) on plasma lipids and white adipose tissues of rats. A total of 18 male albino rats were divided equally into three groups. Each group was fed a diet containing one of these types of oils and fats for 8 weeks. The levels of total cholesterol (TC), high-density lipoprotein cholesterol (HDL-C), low-density lipoprotein cholesterol (LDL-C), total triglyceride (TG), glucose, alanine aminotransferase (ALT), and aspartate aminotransferase (AST) were analyzed in blood plasma. Body mass index (BMI), body weight gain (%BWG), and the weight of adipose tissues were investigated. The results showed no significant differences between groups in TC. However, the highest level of HDL-C was observed in the CHF group, with no significant differences in the PO group and a significant decrease in the CO group. The CHF group showed a significant reduction in LDL-C, blood glucose levels, and the atherosclerosis index compared with the other groups. Furthermore, the lowest TG level was observed in the CHF group, with no significant difference compared with the CO group. The PO group showed a significant decrease in the ALT level compared with the other groups. The lowest AST level was observed in the PO group, with no significant difference compared with the CO group. A significant decrease in the percentage of total adipose tissues, namely epididymal, retroperitoneal, and perirenal cells, was observed in the CHF group. In conclusion, CHF consumption may have a positive effect on plasma lipids and obesity. Moreover, we recommend the completion of research to identify the optimal combination of vegetable oils and CHF for achieving a balance among the health indicators included in this study.

## Introduction

Hyperlipidemia is an important risk factor for cardiovascular disease and a cause of death. It refers to increased cholesterol level, total triglyceride (TG) level, or both (1). Dietary fat affects plasma lipids through its actions to increase or decrease the concentrations of lipoproteins in the blood, especially low-density lipoproteins (LDL-C) and very-low-density lipoproteins (VLDL-C), which are the major transporters of cholesterol and triglycerides (2). Diets containing different types of dietary fat affect cholesterol metabolism in different ways (3). A study of the effects of the intake of saturated and trans-unsaturated fatty acids on mortality and cardiovascular disease showed that saturated fats are not associated with all-cause mortality, cardiovascular disease, and ischemic stroke (4). Another study of the effects of canola oil and palm olein oil (PO) on cardiovascular diseases indicated that using canola oil and PO may increase the risk of atherosclerosis by decreasing paraoxanase-1 (PON1) activity and increasing the level of oxidized low-density lipoproteins (oxLDL) (5). Rats fed a high-fat diet supplemented with pumpkin seed oil showed a decline in body weight gain and altered expression of lipid enzyme indicators, which suggests that pumpkin seed oil may function as an anti-obesity agent by modulating mRNA and enzymes that are important for fat metabolism (6). In a review that focused on identifying the effect of solid fats on blood lipids, beef tallow was found to have a higher influence on reducing LDL-C levels than milk fat (butter) (7). PO consumption in a balanced diet is not a risk factor for cardiovascular disease and atherosclerosis. However, replacing PO with another type of oil that contains monounsaturated and polyunsaturated fatty acids may be less beneficial or non-beneficial (8). Consumption of a high amount of animal fat (butter) may cause hypercholesterolemia, whereas the consumption of plant fat sources (soybean oil) has a positive effect on plasma lipids, body weight, and blood glucose (9).

In addition, the composition of fatty acids in different types of fat and oil may affect plasma lipids and obesity. Den Hartigh (10) reported that conjugated linoleic acid (CLA) may exert a positive effect on cancer, atherosclerosis, and obesity. Palmitic acid has an influence on the reduction of plasma and liver triglyceride levels (11). Unsaturated fatty acids can have a positive effect on triglyceride levels and increase high-density lipoprotein levels (12). Farina observed that linoleic acid does not affect plasma triglyceride levels but influences fat storage and increases lipogenic enzyme activity and mRNA levels involved in fatty acid production (13). Moreover, soluble vitamins in oils and fats may have a positive effect on cardiovascular markers and obesity (14–18).

In the literature, some studies have reported the positive effect of animal fats on blood lipid profile, body weight, and blood glucose, whereas others have reported the opposite. Therefore, this study aimed to investigate the effect of CHF as an animal and saturated fatty acid source and of PO and CO as plant oil sources and also monounsaturated and polyunsaturated fatty acid sources, respectively.

## Material and method

### Animals and treatment

This experiment was approved by the Research Ethics Committee of the Deanship of Scientific Research, King Faisal University, Saudi Arabia (reference number: KFU-REC/2020-04-22).

The experiment was conducted in the College of Agricultural and Food Sciences at King Faisal University, Al-Ahsa, Saudi Arabia. A total of 18 male albino rats aged 4 weeks and ranging from 80 to 100 g of body weight were equally divided into three groups: rats fed a diet containing camel hump fat (CHF), those fed a diet containing corn oil (CO), and those fed a diet containing palm olein oil (PO). Rats were acclimated for 1 week under standard laboratory conditions (temperature, 20 c; humidity, 40%). Each group was fed the experimental diet for 8 weeks with access to food and water. Diet compositions are presented in Table 1. Following a previous study, diets were prepared with a modification to the fat percentage (19). Six rats from each group were kept in a cage and given 200 g of feed and 500 mL of water. The next day, at the same time, the remaining feed was weighed, the amount consumed was calculated, and the average feed consumption per rat for that day was calculated by dividing the amount consumed by the number of rats. These steps were followed for 7 consecutive days in the first week of the experiment and also in the last week of the experiment. Then, the average food intake for the 7 days in the first and last weeks was calculated. All rats were weighed at the end of each week to calculate body mass index (BMI) and body weight gain (%BWG). After the end of each week, the origin tissues (heart, spleen, kidneys, and liver) of eight rats were dissected, collected, and weighed, and the livers were kept in saline solution at −18°C. In addition, white adipose tissues were collected, weighed, and kept in 10% formalin for histological analysis.

**Table 1:** Diet composition (g/100 g).

Ingredient	CHF	CO	PO
Starch	50	50	50
Casein	15	15	15
Oil or fat	20	20	20
Cellulose	10	10	10
Minerals mixture	4	4	4
Vitamins mixture	1	1	1

PO, Palm olein oil; CO, corn oil, and CHF, camel hump fat.

### Diet composition

Casein powder (Fonterra, New Zealand), starch (Middle East Food Solutions, KSA), cellulose (Nutricology, Alameda California), mineral mixer (Silex grit), and vitamin mixer (AIN-76) were purchased from the local market.

### Chemical properties of different types of oil and fat

Palm olein oil and corn oil were purchased from the local market. CHF was prepared by purchasing the camel hump from the meat market, melting it by heating, and then filtering the fat.

Fatty acid profiles of the different types of fat and oil were determined using the GC AOAC-996.01 method (20). The levels of vitamins K, E, D, and A were determined using a spectrophotometric method, as described in a previous study (21, 22). The levels of β-carotene and chlorophyll pigments were determined using a spectrophotometric method, as described by Munasinghe and Wansapala (23) and Ward et al. (24), respectively. Iodine values and saponification values were determined using titration methods, as described by Davies and Boley (25). Acid values and peroxidase values were determined using titration methods, as described by Kirk and Sawyer (26).

### Blood collection

After subjecting all rats to fasting for 8 h, 1.5 mL of blood was collected from their eyes using a sodium heparinized capillary tube in an Eppendorf tube containing EDTA. The same procedure was repeated after 8 weeks of feeding on different experimental diets. Blood samples were centrifuged at 3,000 rpm for 18 min to acquire blood plasma and kept in the freezer at −18°C.

### Determination of plasma parameters

The levels of plasma total cholesterol (TC), high-density lipoprotein cholesterol (HDL-C), total triglyceride (TG), plasma glucose, alanine aminotransferase (ALT), and aspartate aminotransferase (AST) were directly assayed using kits (Quimica Clinica Aplicada, Spain) and a spectrophotometer (UV1800, Japan) (27–29). Low-density lipoprotein cholesterol levels (LDL-C) and atherosclerosis index were also calculated (19):


LDL−C&VLDL−C(mg/dl)=TC–HDL−C.

Atherosclerosis index=LDL−C/HDL−C.

### Body mass index (BMI) and body weight gain (%BWG)

BMI and %BWG were calculated as described in a previous study (Abd (19)):


$$\mathrm{BMI}=\left(\mathrm{final weight}\right)/\left({\mathrm{length}}^{2}\right).$$

%BWG=((final weight–initial weight)/(initial weight))∗100.

### Liver TC and TG

Frozen livers in the saline solution were milled, each liver was transferred to a plastic bag, and the samples were mashed. Of each sample, 0.1 g was weighed in a test tube with a stopper cover, and 10 mL of a solution containing chloroform and methanol (2:1) was added. The test tube was covered, shaken, and filtered using a filter paper in an Eppendorf tube. The samples are then analyzed in the same way as for TC and TG in blood plasma (30).

### Histological analysis

White adipose tissues were treated with a tissue processor and embedded in paraffin. Tissues were sectioned into 4 μm sections using a rotary microtome (Leica microsystem, Germany) and stained using hematoxylin and eosin. The stained tissues were observed using an Olympus BX51 photomicroscope (Olympus Inc., Japan) at 200x magnification. The area of 100 adipose cells from stained adipose tissues in each group was measured using Optika Pro View software (31, 32, 47).

### Statistical analysis

Data were analyzed using SAS 9.0 software (SAS Institute) and expressed as mean ± standard deviation. All data were analyzed based on a completely randomized design using a one-way analysis (ANOVA). Differences between means were evaluated using a least significant differences (LSD) test to evaluate significant differences, and a *p*-value of <0.05 was considered statistically significant.

## Results

### Chemical properties of different types of oil and fat

As shown in Table 2, the predominant fatty acids in CHF were saturated fatty acids, composed of palmitic acid (C16:0) and stearic acid (C18:0), and monounsaturated fatty acids, primarily oleic acid (C18:1). In addition, the predominant fatty acids in CO were unsaturated fatty acids, composed of linoleic acid (C18:2) and oleic acid (C18:1). Furthermore, the predominant fatty acids in PO were monounsaturated fatty acids, primarily oleic acid (C18:1), and saturated fatty acids, primarily palmitic acid (C16:0).

**Table 2:** Fatty acid composition, vitamins, and pigments of different types of fat and oil.

Fatty acids (%)	CHF	CO	PO
C4:0 (butryic) C4H8O2	–	–	–
C6:0 (caproic) C6H12O2	–	–	–
C8:0 (caprylic) C8H16O2	–	–	0.02
C10:0 (capric)C₁₀H₂₀O₂	0.01	–	0.02
C11:0 (undecanoic)C₁₁H₂₂O₂	–	–	–
C12:0 (lauric)C₁₂H₂₄O₂	0.42	–	0.24
C13:0 (tridecanoic)C₁₃H₂₆O₂	0.07	–	-
C14:0 (myristic)C₁₄H₂₈O₂	5.47	0.02	0.98
C14:0 (myristoleic)C₁₄H₂₆O₂	0.11	–	–
C15:0 (pentadecanoic)C₁₅H₃₀O₂	1.58	–	0.04
C15:1 (cis-10-pentadecanoic)	–	–	-
C16:0 (palmitic)C₁₆H₃₂O₂	36.24	11.34	39.87
C16:1 (palmitoleic)C₁₆H₃₀O₂	3.97	0.08	0.16
C17:0 (heptadecanoic)C₁₇H₃₄O₂	1.42	0.06	0.08
C17:1 (cis-10Heptadecanoic)	0.76	0.02	0.02
C18:0 (stearic)C₁₈H₃₆O₂	15.11	1.50	4.02
C18:1n9t (elaidic)C₁₈H₃₄O₂	2.64	–	–
C18:1n9c (oleic)C₁₈H₃₄O₂	28.46	28.48	42.95
C18:2n6t (linolelaidic)C₁₈H₃₂O₂	0.02	–	–
C18:2n6c(linoleic)C₁₈H₃₀O₂	1.54	56.78	10.70
C20:0 (arachidic)C₂₀H₄₀O₂	0.30	0.33	0.34
C18:3n6 (Y-linolenic)C₁₈H₃₀O₂	0.04	0.02	0.03
C20:1n9 (cis-11-eicosenoic)C₂₀H₃₈O₂	0.47	0.21	0.18
C18:3n3 (A-linolenic)C₁₈H₃₀O₂	0.23	0.88	0.19
C21:0 (heneicosanoic)C₂₁H₄₂O₂	0.93	–	–
C20:2 (cis-11,14-eicosadienoic)C₂₀H₃₆O₂	0.02	0.02	-
C22:0 (behenic)C₂₂H₄₄O₂	0.04	0.10	0.06
C20:3n6 (cis-8,11,14-eicosatrienoic)C₂₀H₃₄O₂	0.03	–	–
C22:1n9 (erucic)C₂₂H₄₂O₂	0.04	–	–
C20:3n3 (cis-11,14,17-eicosatrienoic) C20H34O2	0.02	–	–
C23:0 (tricosanoic)C₂₃H₄₆O₂	0.04	–	–
C20:4n6 (arachidonic)acid C₂₀H₃₂O₂	–	0.01	–
C22:2 (cis-13,16-docosadienoic)C₂₂H₄₀O₂	–	–	–
C24:0 (lignoceric)C₂₄H₄₈O₂	–	0.14	0.07
C20:5n3 (cis-eicosapentaenoic acid)C₂₀H₃₀O₂	–	–	–
C24:1n9 (nervonic) C24H46O2	0.02	–	–
Saturated fatty acids	61.58	13.48	45.70
Unsaturated fatty acids	38.34	86.47	54.18
Monounsaturated fatty acids	33.80	28.78	43.27
Polyunsaturated fatty acids	1.88	57.69	10.91
Trans-fatty acids	2.66	<LOQ	<LOQ
Vitamin A (μg/g)	81.52 ± 9.86^ab^	76.5 ± 9.02^b^	98.18 ± 5.48^a^
Vitamin D (IU/g)	4924.92 ± 1801.39	5160.97 ± 1281.02	5281.57 ± 644.57
Vitamin E (mg/kg)	47.5059 ± 2.13	47.5120 ± 2.32	47.5146 ± 9.29
Vitamin K (mg/g)	12.12 ± 0.05	12.174 ± 0.02	12.637 ± 0.67
β-Carotene (mg/kg)	15.71 ± 2.59^b^	17.28 ± 3.54^ab^	21.21 ± 0.26^a^
Chlorophyll (mg/kg)	-	11.05 ± 1.65	-

Fatty acids denoted by (–) are undetectable. Vitamin and pigment levels are shown as mean ± SD on *n* = 3 replicate per sample. Values in the same row with different superscript letters are significantly different, *p* < 0.05. PO, Palm olein oil; CO, corn oil; CHF, camel hump fat.

The content of vitamins and pigments soluble in fats and oils used in the experiment is shown in Table 2. The highest content of vitamin A was observed in PO, with no significant difference observed between PO and CHF, and the lowest content was observed in CO, with no significant difference compared with CHF. No significant difference was observed in the content of vitamins K, E, and D among the PO, CO, and CHF groups. The highest *β*-carotene content was observed in PO, with no significant difference compared with CO, and the lowest PO content was observed in the CHF group. The presence of chlorophyll pigment was observed only in CO.

The chemical properties of different types of oil and fat, namely iodine value, acid value, saponification value, and peroxide value, are shown in Table 3. CO showed the highest iodine value, which was significantly differing from the other types. The high iodine value is due to the fact that CO contains a high percentage of polyunsaturated fatty acids. The highest saponification value was observed in CHF, showing significant differences compared with the other types of oils, while CO and PO exhibited no significant difference in saponification value. High saponification values indicate a high percentage of short fatty acid chains in oils and fats. The acid value of CHF was significantly higher than that of CO and PO, which is likely due to CO and PO being refined oils. CHF showed a lower peroxide value than CO and PO as it contains a high amount of saturated fatty acids, which are more stable to oxidation. The highest peroxide value was observed in CO, with significant differences compared with PO, as it contains a high percentage of unsaturated fatty acids, as shown in Table 2. However, all oil and fat types had acid values and peroxide values within the acceptable range according to the Gulf Standards organization.

**Table 3:** Chemical properties of different types of oil and fat.

Parameter	CHF	CO	PO
Iodine value (gI/100 g)	70.23 ± 6.00^b^	117.79 ± 10.62^a^	76.93 ± 2.22^b^
Acid value (mg KOH/g)	0.41 ± 0.00^a^	0.13 ± 0.02^c^	0.33 ± 0.03^b^
Saponification value (mg KOH/g)	197.75 ± 0.42^a^	185.62 ± 4.81^b^	188.54 ± 2.52^b^
Peroxide value (mEq/Kg)	0.25 ± 0.00^c^	1.96 ± 0.01^a^	1.33 ± 0.08^b^

Data are shown as mean ± SD on *n* = 3 replicate per sample. Values in the same row with different superscript letters are significantly different, *p* < 0.05. PO, Palm olein oil; CO, corn oil; CHF, camel hump fat.

### Effect of feeding on different types of oil and fat on plasma parameters

The levels of different plasma parameters assessed in this study in rats fed on different types of dietary fat and oils for 8 weeks are shown in Table 4. These results showed no significant differences in the plasma TC level between groups. However, the highest levels of plasma HDL-C were observed in the CHF group, with no significant difference compared with the PO group, while the lowest HDL-C levels were observed in the CO group, with significant difference compared with the CHF or PO groups. Moreover, the lowest plasma LDL-C levels were observed in the CHF group, with significant differences compared with the CO and PO groups. The results indicate no significant differences in the plasma TG levels between the CHF group and the PO group, and the highest plasma TG levels were observed in the CO group, with significant differences compared with the CHF and PO groups. In addition, rats in the CHF group showed the lowest atherosclerosis index, with a significant difference compared with the PO and CO groups, and no significant differences were observed between the PO group and the CO group. However, the CHF group showed low plasma glucose levels, with significant differences compared with the other groups. The lowest ALT and AST levels were observed in the PO group, with significant differences compared with the CHF group. The low values may be due to PO having a high vitamin A and *β*-carotene content, which prevents damage to the liver and kidneys (33).

**Table 4:** Effects of feeding on different types of oil and fat on plasma parameters.

Parameter	Beginning	Group		
		CHF	CO	PO
TC mg/dl	73.8 ± 0.63	86.84 ± 0.96	85.81 ± 7.75	88.54 ± 4.22
HDL-C mg/dl	46.91 ± 0.8	60.00 ± 5.11^a^	53.63 ± 5.35^b^	55.27 ± 3.35^ab^
LDL-C and VLDL-C mg/dl	26.89 ± 1.44	26.84 ± 6.07^b^	32.17 ± 2.76^a^	33.27 ± 2.90^a^
Atherosclerosis index	0.58 ± 0.03	0.45 ± 0.14^b^	0.60 ± 0.03^a^	0.60 ± 0.06^a^
TG mg/dl	32.40 ± 0.83	35.58 ± 1.34^b^	35.60 ± 5.09^b^	49.31 ± 1.00^a^
Glucose mg/dl	101.60 ± 0.19	130.45 ± 6.36^b^	154.04 ± 1.35^a^	150.17 ± 3.66^a^
ALT U/L	20.30 ± 0.57	33.73 ± 5.09^a^	31.99 ± 3.82^ab^	27.62 ± 4.77^b^
AST U/L	47.82 ± 6.13	49.73 ± 5.00^b^	64.56 ± 7.46^a^	38.39 ± 4.46^c^

Data are shown as mean ± SD on *n* = 6 replicate per group. Values in the same row with different superscript letters are significantly different, *p* < 0.05. PO, Palm olein oil; CO, corn oil; CHF, camel hump fat.

### Effects of feeding on different types of oil and fat on body weight, %BWG, and BMI

The results and differences between groups regarding weight, food intake, BMI, %BWG, organ weight, adipose tissue weight, and liver lipids are shown in Table 5. No significant differences in food intake were observed between groups at the baseline or after 8 weeks of feeding. The mean initial weight of the PO, CO, and CHF groups was 89.5, 91.00, and 86.00, respectively. However, there were no significant differences between groups in the initial weight. At the end of the experiment, the PO group recorded the highest final weight, and the CHF group recorded the lowest.

**Table 5:** Effects of feeding on different types of oil and fat on body weight, BMI, %BWG, origin weight, adipose tissues, and liver lipids.

Parameter	Group		
	CHF	CO	PO
Initial weight (g)	86.00 ± 4.38	91.00 ± 8.76	89.5 ± 8.21
Final weight (g)	258.0 ± 38.34^b^	305.5 ± 39.98^a^	315.0 ± 18.62^a^
Food intake in the beginning (g)	13.35 ± 0.80	13.35 ± 0.74	13.28 ± 0.99
Food intake after 8 weeks (g)	18.78 ± 0.56	18.64 ± 0.80	18.57 ± 0.67
BMI	0.52 ± 0.00^c^	0.74 ± 0.02^a^	0.59 ± 0.00^b^
%BWG	229.92 ± 27.38	276.95 ± 35.08	280.65 ± 24.22
% Heart	0.35 ± 0.03	0.38 ± 0.13	0.37 ± 0.09
% Liver	4.00 ± 0.08	4.13 ± 0.91	3.98 ± 0.94
% Kidney	0.67 ± 0.08	0.62 ± 0.16	0.70 ± 0.10
% Spleen	0.32 ± 0.00^a^	0.22 ± 0.03^c^	0.27 ± 0.02^b^
% Total adipose tissues	1.56 ± 0.02^b^	2.50 ± 0.92^a^	1.83 ± 0.36^ab^
% Epididymal	0.36 ± 0.05^b^	0.51 ± 0.10^a^	0.46 ± 0.11^ab^
% Mesenteric	0.80 ± 0.00	0.97 ± 0.00	0.76 ± 0.06
% Retroperitoneal	0.08 ± 0.01^b^	0.22 ± 0.08^a^	0.08 ± 0.02^b^
% Perirenal	0.31 ± 0.02^b^	0.79 ± 0.40^a^	0.50 ± 0.07^ab^
Liver TC (mg/g)	6.88 ± 1.50^b^	11.00 ± 4.01^b^	22.93 ± 7.03^a^
Liver TG (mg/g)	29.29 ± 4.39^a^	22.92 ± 0.00^b^	27.38 ± 3.48^a^

Data are shown as mean ± SD. Values in the same row with different superscript letters are significantly different between groups, *p* < 0.05. PO, Palm olein oil; CO, corn oil; CHF, camel hump fat.

No significant differences in %BWG were observed between groups, while the highest BMI was observed in the CO group and the lowest in the CHF group. However, while a normal BMI was observed in the PO and CHF groups, the CO group showed a higher-than-normal BMI Mamikutty et al. (34) and Novelli et al. (35) showed that normal BMI in rats is between 0.45 and 0.68 g/cm^2^. However, BMI is an inaccurate indicator of obesity in animals and humans, whereas the percentage of fat tissues is a more accurate indicator (36).

As shown in Table 5, the highest percentage of total adipose tissues was observed in the CO group, with no significant differences observed compared with the PO group. The lowest percentage of total adipose tissues was observed in the CHF group, with a significant difference compared with the CO group. However, there are no significant differences in the percentage of total adipose tissues between the PO group and the CHF group.

### Adipose tissue cell area

**Figure 1:**
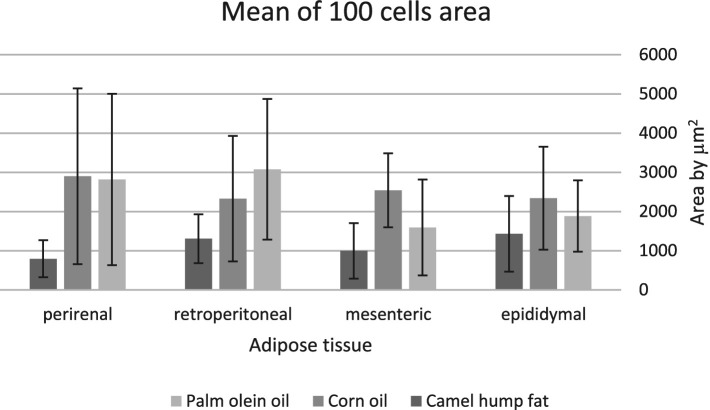
Mean area (μm^2^) of 100 cells of different types of adipose tissue.

**Figure 2:**
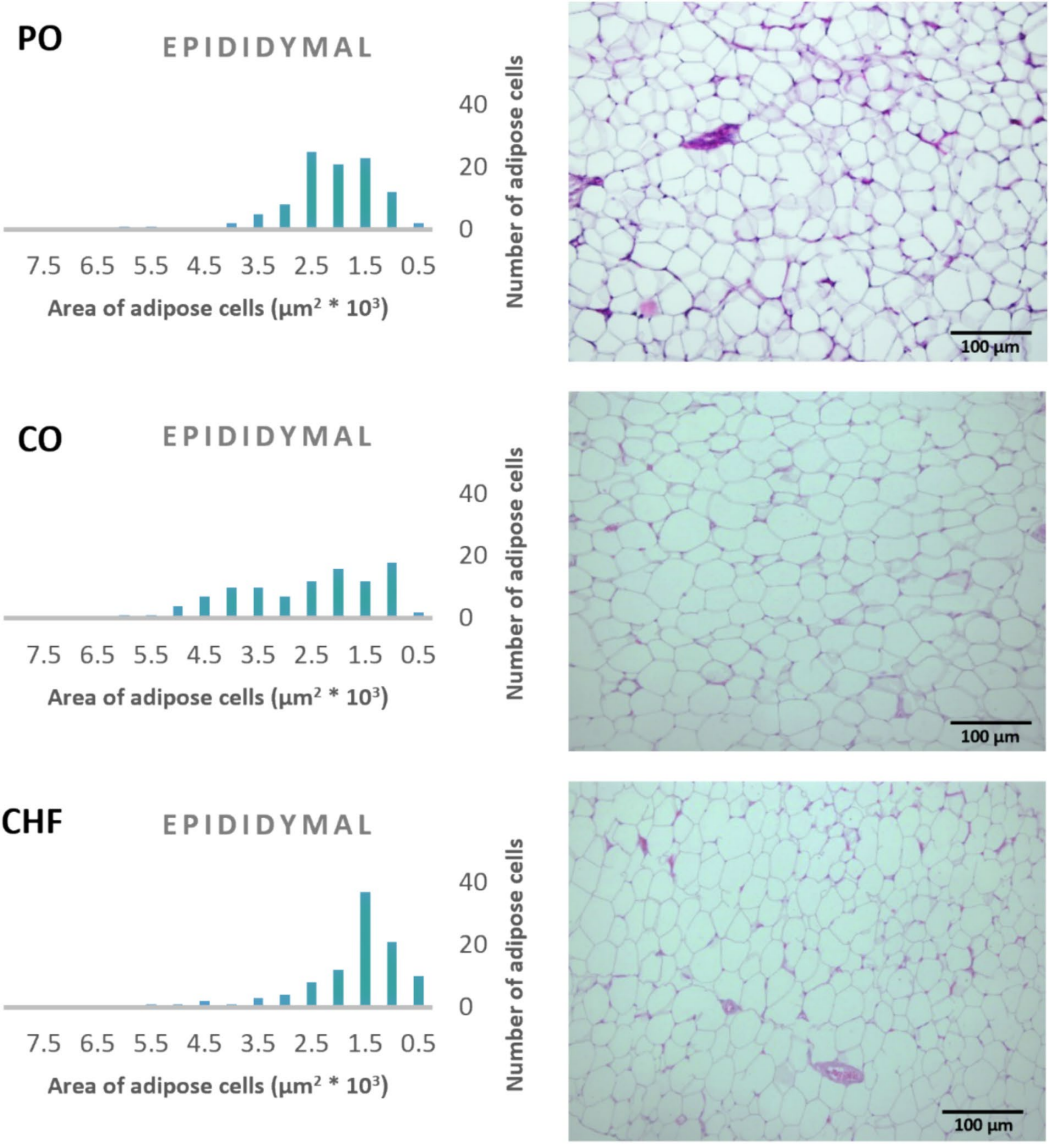
Effects of different types of dietary fat and oil on cell area of adipose tissue (epididymal). PO, Palm olein oil; CO, corn oil; CHF, camel hump fat.

**Figure 3:**
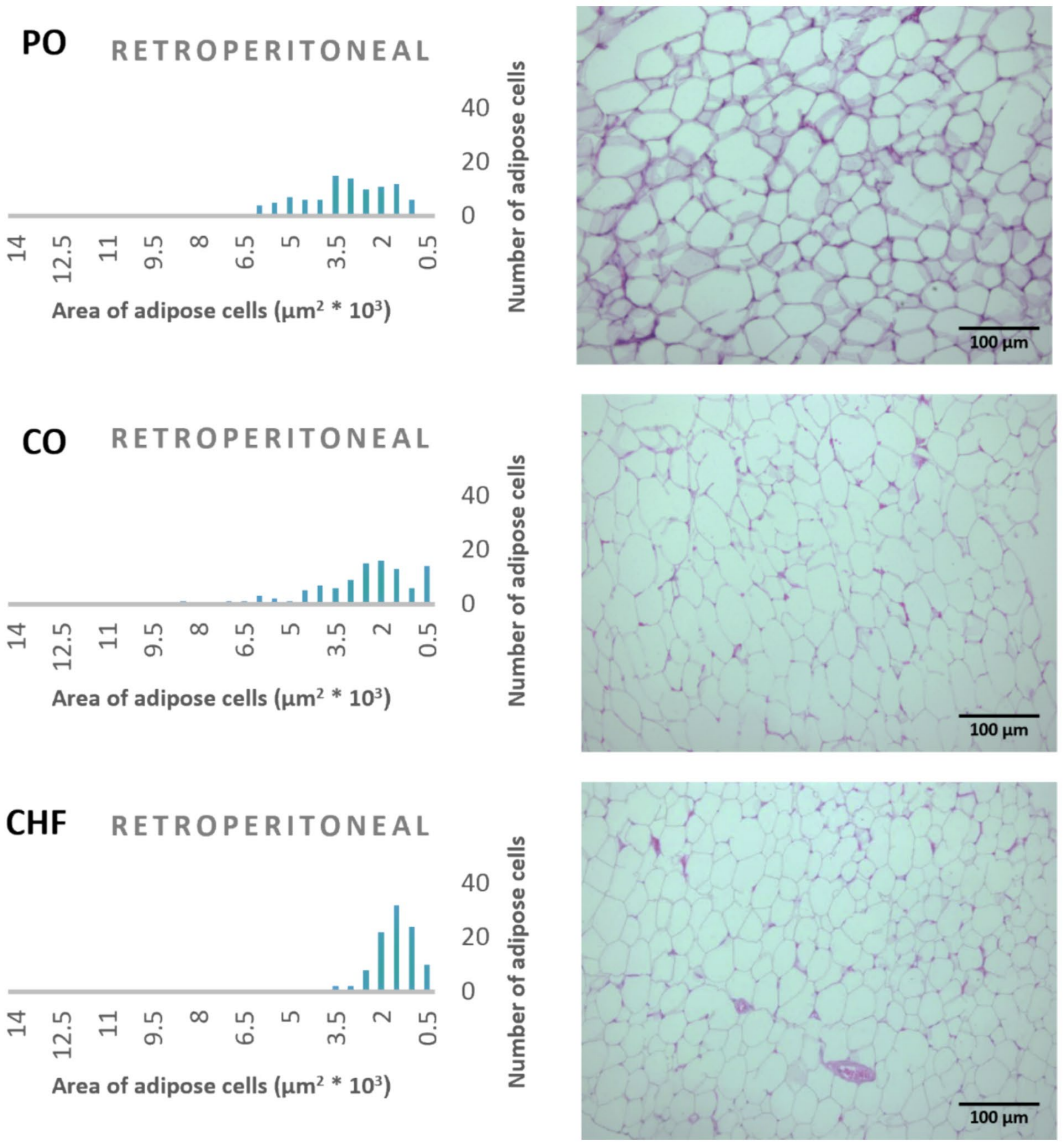
Effects of different types of dietary fat and oil on cell area of adipose tissue (retroperitoneal). PO, Palm olein oil; CO, corn oil; CHF, camel hump fat.

**Figure 4:**
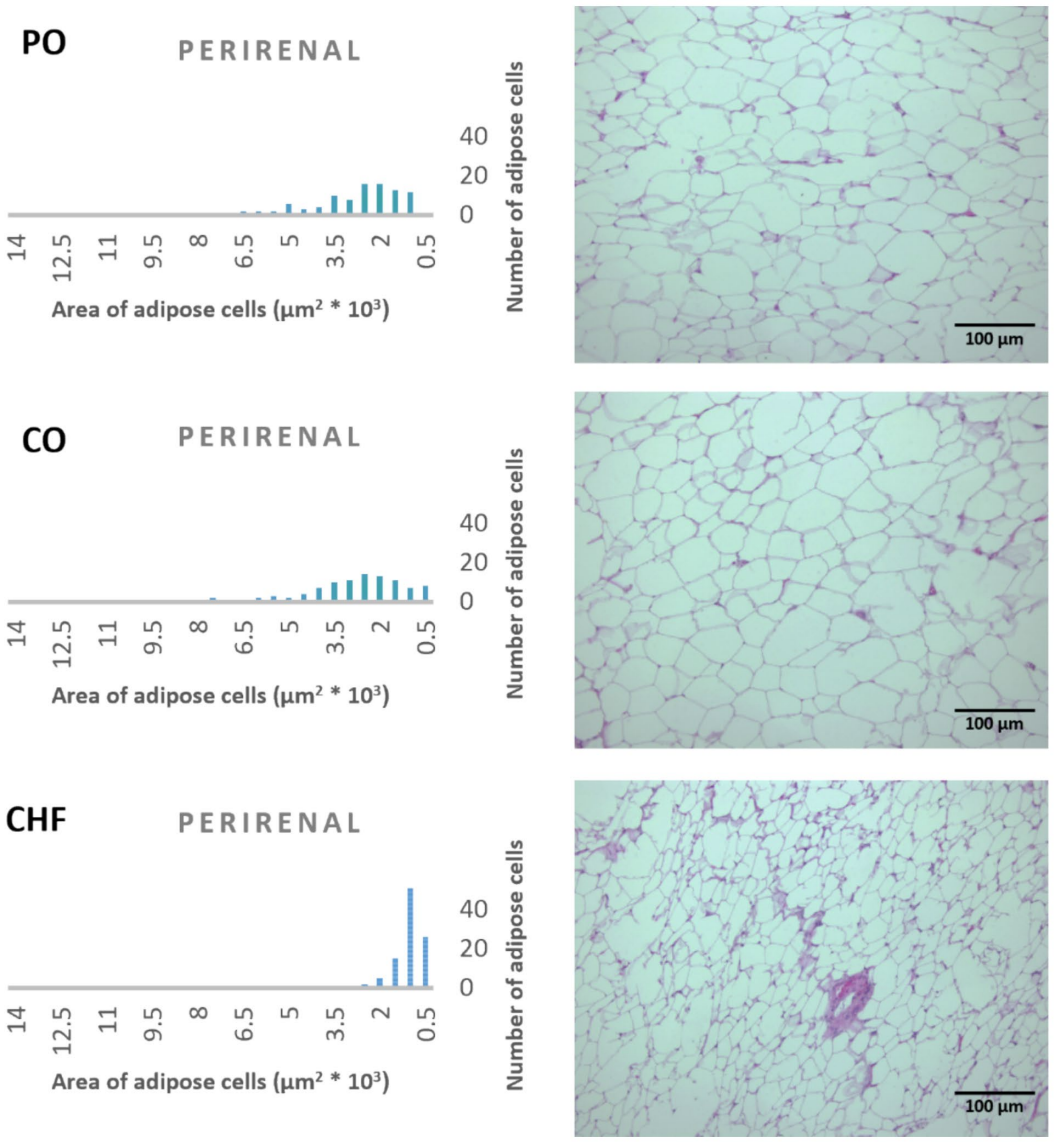
Effects of different types of dietary fat and oil on cell area of adipose tissue (perirenal). PO, Palm olein oil; CO, corn oil; CHF, camel hump fat.

**Figure 5:**
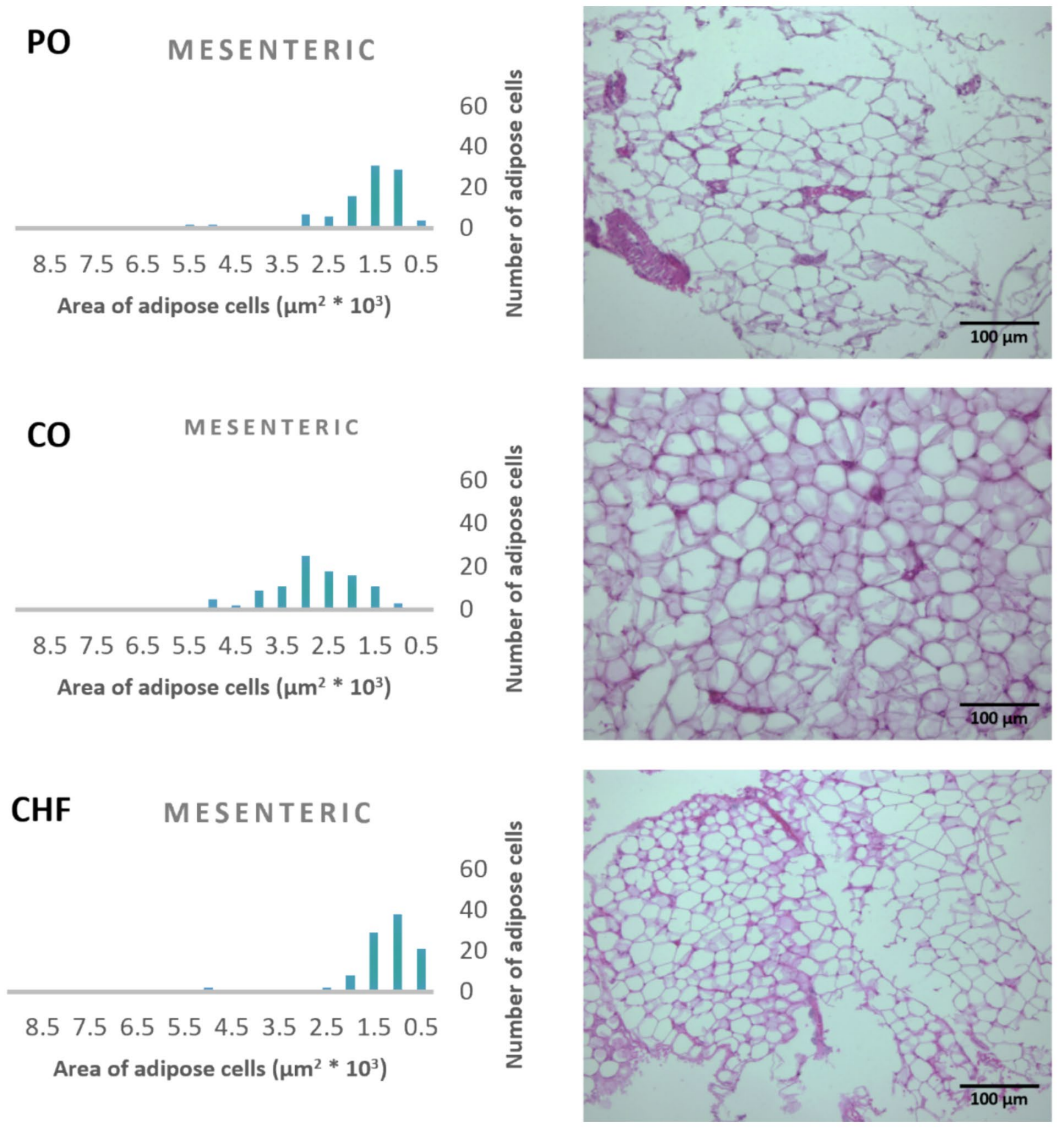
Effects of different types of dietary fat and oil on cell area of adipose tissue (mesenteric). PO, Palm olein oil; CO, corn oil; CHF, camel hump fat.

The average area of 100 adipose tissue cells in different types of adipose tissues (namely epididymal, mesenteric, retroperitoneal, and perirenal) is shown in Figure 1. CHF reduced the area of the adipose cells in epididymal cells, with a significant difference compared with the other types of oil (Figures 1, 2). However, as shown in Figures 1, 2, CO increased the area of epididymal cells, with a significant difference compared with PO and CHF. In addition, the highest average area of 100 adipose tissue in the mesenteric cells was observed in the CO group, with significant differences compared with the other groups, whereas the lowest average was observed in the CHF group. The effects of different types of dietary fat and oil on retroperitoneal cells are shown in Figure 3. Numerous small cells were observed in the CHF group, and the lowest number of small cells was observed in the PO group. The highest average area of 100 perirenal cells was observed in the CO group, with no significant differences compared with the PO group, and the lowest area was observed in the CHF group, with a significant difference compared with other groups. In addition, the highest number of small perirenal cells was observed in the CHF group (Figure 4). As shown in Figure 5, a higher number of large mesenteric cells was observed in the CO group, and the lowest number of large cells was observed in the CHF group. PO increased the average area of retroperitoneal cells, with significant differences compared with the CO and CHF groups. The CHF group showed a significant decrease in the average area of retroperitoneal cells compared with the CO and PO groups.

There were no significant differences between groups in the percentage of heart, liver, and kidney attributes to final body weight. A significant increase in spleen percentage was observed in the CHF group, while the lowest spleen percentage was observed in the CO group.

## Discussion

A previous study reported a significant increase in serum TC levels in rats fed PO compared with rats fed other types of oil (5). This increase may be due to saturated fatty acids, as Baudet et al. (37) showed that high consumption of saturated fatty acids can lead to an increase in blood TC levels. In our study, the lowest levels of HDL-C were observed in the CO group, and the highest levels were observed in the CHF group. In addition, Rezq et al. (38) reported no significant difference in serum HDL-C levels in mice fed CO or milk fat (butter). Amini et al. (5) showed a significant increase in blood TG levels of rats fed on a diet containing PO compared with groups that fed on a hydrogenated PO diet and the control group. Our results are in agreement with these results; we observed an increase in the blood TG levels in the PO group and low levels of blood TG levels in the CHF and CO groups. However, the positive effects of CHF on blood lipids may be due to the amount of palmitoleic acid in CHF (Table 2). Palmitoleic acid may have the ability to reduce the development of atherosclerosis, improve fat and glucose metabolism, and have a positive effect on fat production (39). Bi et al. (40) showed that oleic acid and stearic acid may have a positive effect on blood TC and TG levels, while linoleic acid may have a negative effect on blood TC and TG levels. In addition, a mice diet free from linoleic acid was shown to be associated with an increase in lipoprotein lipase activity; furthermore, linoleic acid is involved in increasing lipogenic enzyme activity and high levels of mRNA, which both play a role in fat production (13). Mice fed only lard for 12 weeks showed the lowest ALT level compared with those fed sunflower oil and a mixture of lard and sunflower oil. In our results, no significant differences were observed between the CHF and CO groups (41). Our results are in agreement with those of Yan et al. (41); they reported that mice fed lard showed a significant reduction in ALT levels compared with those fed sunflower oil. However, the positive effect of PO on ALT and AST levels may be due to the high amount of vitamins A, D, E and K and *β*-carotene in PO. Owu et al. (33) reported that free radicals destroy liver cells, leading to an increase in these enzymes. However, when feeding on antioxidant sources, the rate of liver cells destruction decreases, and resulting in a decrease in enzyme levels.

Boon et al. (42) showed that rats fed PO for 15 weeks recorded a significant increase in final body weight compared with the control group. However, Yan et al. (41) reported that mice fed animal fat (lard) showed a lower final body weight than those fed sunflower oil and a mixture of sunflower oil and lard.

Buchan et al. (43) showed that the weight of the spleen decreases with increased movement. Sundram et al. (44) showed that there are no significant differences in the weight of heart, liver, kidney, and spleen between rats fed PO and rats fed CO for 15 weeks. Furthermore, Kritchevsky et al. (11) reported that there was no significant difference in liver percentage between rats fed PO and those fed CO for 3 weeks.

Our results are consistent with those of Inai and Matsuo (3), who showed no significant differences in liver TC and liver TG levels between rats fed CO and those fed beef tallow (animal fat source). While Kritchevsky et al. (11) observed no significant difference in liver TC levels between feeding on CO and PO for 3 weeks, CO caused a significant increase in liver TG levels compared with PO, and these results disagree with ours.

Yan et al. (41) showed that rats fed lard (animal fat) for 12 weeks showed a lower weight of epididymal, retroperitoneal, and total adipose tissues than those fed sunflower oil and a mixture of lard and sunflower oil. Pavlisova et al. (45) observed a significant increase in epididymal weight in mice fed CO for 8 weeks compared with the control group. Moreover, mice fed PO for 15 weeks showed a significant decrease in the total adipose tissue weight compared with mice fed olive oil for 15 weeks (Sin (46)). BMI is often an inaccurate indicator of obesity, whereas the percentage of body fat is the best indicator (36). However, in the present study, the CHF group showed the lowest BMI and the lowest percentage of total adipose tissue, and there was no significant difference between groups in the amount of food consumed at either the beginning or the end of the experiment. The CHF group also showed the lowest %BWG. It can be concluded that the consumption of CHF led to a reduction in weight gain compared with other oils under the same conditions and with approximately the same amount of feed. The positive effect of CHF on white adipose tissue may be due to increased hydrolysis of triglycerides and increased rate of β-oxidation of fatty acids and inhibition of fat synthesis. In addition, stearic fatty acid position may affect Yan et al. (41) described that the stearic fatty acid in the third position in the triglyceride may undergo hydrolysis to the triglyceride and fatty acid linking with Ca^+^ or Mg^+^ to form an insoluble salt, and it may reduce fat absorption and contribute to reducing adipose tissues weight.

Our results are consistent with those of Yan et al. (41) mice fed lard showed the lowest epididymal cell area than those fed sunflower oil and those fed a mixture of lard and sunflower oil for 12 weeks.

## Conclusion

Consuming CHF may have a positive effect on plasma lipids and obesity compared with CO and PO. No effect was observed on total plasma cholesterol in all treatments, while the highest level of HDL-C was observed in the CHF group. The same group showed the lowest levels of LDL-C, triglycerides, and blood glucose, and the lowest atherosclerosis index. In addition, the PO group showed the lowest levels of AST and ALT. A diet containing CHF reduces the percentage of epididymal, perirenal, and retroperitoneal cells, with significant differences compared with a diet containing CO. We recommended the completion of research to identify the optimal combinations of vegetable oils and CHF for achieving a balance among the health indicators included in this study.

## Data Availability

The original contributions presented in the study are included in the article/supplementary material, further inquiries can be directed to the corresponding author.
